# Inflammatory pathways are upregulated in the nasal epithelium in patients with idiopathic pulmonary fibrosis

**DOI:** 10.1186/s12931-018-0932-7

**Published:** 2018-11-26

**Authors:** Marc A. Sala, Yalbi Itzel Balderas-Martínez, Ivette Buendía-Roldan, Hiam Abdala-Valencia, Kiwon Nam, Manu Jain, Sangeeta Bhorade, Ankit Bharat, Paul A. Reyfman, Karen M. Ridge, Annie Pardo, Jacob I. Sznajder, G. R. Scott Budinger, Alexander V. Misharin, Moises Selman

**Affiliations:** 1 Division of Pulmonary and Critical Care Medicine, Feinberg School of Medicine, Northwestern University, Chicago, IL USA; 2 CONACYT–Instituto Nacional de Enfermedades Respiratorias Ismael Cosío Villegas, Mexico City, Mexico; 3 Instituto Nacional de Enfermedades Respiratorias Ismael Cosío Villegas, Tlalpan 4502, CP 14080 Mexico City, Mexico; 4 Facultad de Ciencias, Universidad Nacional Autónoma de Mexico, Mexico City, Mexico

**Keywords:** Pulmonary fibrosis, Nasal transcriptome, Immune response, Virus, Bacteria

## Abstract

Idiopathic pulmonary fibrosis (IPF) is characterized by progressive scarring of the lung parenchyma, leading to respiratory failure and death. High resolution computed tomography of the chest is often diagnostic for IPF, but its cost and the risk of radiation exposure limit its use as a screening tool even in patients at high risk for the disease. In patients with lung cancer, investigators have detected transcriptional signatures of disease in airway and nasal epithelial cells distal to the site of disease that are clinically useful as screening tools. Here we assessed the feasibility of distinguishing patients with IPF from age-matched controls through transcriptomic profiling of nasal epithelial curettage samples, which can be safely and repeatedly sampled over the course of a patient’s illness. We recruited 10 patients with IPF and 23 age-matched healthy control subjects. Using 3′ messenger RNA sequencing (mRNA-seq), we identified 224 differentially expressed genes, most of which were upregulated in patients with IPF compared with controls. Pathway enrichment analysis revealed upregulation of pathways related to immune response and inflammatory signaling in IPF patients compared with controls. These findings support the concept that fibrosis is associated with upregulation of inflammatory pathways across the respiratory epithelium with possible implications for disease detection and pathobiology.

**Electronic supplementary material:**

The online version of this article (10.1186/s12931-018-0932-7) contains supplementary material, which is available to authorized users.

## Introduction

Idiopathic pulmonary fibrosis (IPF) is an age-related, chronic, progressive, and usually lethal fibrosing interstitial pneumonia of unknown etiology [1]. The pathogenic mechanisms have not been elucidated, but a growing body of evidence suggests that the convergence of genetic susceptibility, accelerated lung aging, and a profibrotic epigenetic reprogramming provoke an aberrant activation of the lung epithelium and consequently the expansion and activation of the fibroblast/myofibroblast population and the recruitment of profibrotic macrophages that lead to the exaggerated accumulation of extracellular matrix [2–4].

Currently, high resolution computed tomography (HRCT) of the chest is the only non-invasive tool available to screen for the presence of IPF [1]. Widespread HRCT screening for pulmonary fibrosis is not feasible given the relatively low disease prevalence, the high cost of HRCT scanning, and the risk of radiation exposure. As a result, the diagnosis of IPF is often delayed until patients have advanced, functionally limiting disease. Studies of serum biomarkers and transcriptomic profiling of peripheral blood mononuclear cells have failed to identify biomarkers or gene signatures with sufficient sensitivity for screening [5]. Accordingly, there is an unmet clinical need for biomarkers that can be used to identify patients and define disease endotypes that guide clinical therapy.

Studies of the transcriptional signature in the IPF lungs have shown that the disease is characterized by the upregulation of several matrix metalloproteinases, extracellular matrix proteins, molecules involved in developmental pathways, growth factors, and epithelial-related genes such as cytokeratins, mucin-5B, and desmoplakin [6–11]. These studies require access to fibrotic lung tissue from alveolar biopsies, limiting their utility for screening or disease management. In patients with lung cancer, Spira and colleagues observed transcriptomic changes associated with smoking injury and malignancy in respiratory epithelial tissues from the bronchi [12]. They found that a composite score based on the expression of 11 of these genes was sufficiently sensitive to aid in the management of small asymptomatic nodules in the distal lung detected by computed tomography screening, and this gene signature is now FDA approved for use in clinical practice. Since publication of these results, the Spira group has gone on to show that a similar signature can be detected in transcriptomes obtained by nasal epithelial curettage, opening the possibility for a truly non-invasive test to inform the management of suspected cancer in the distant lung [13, 14]. While less well studied, transcriptomic analysis of lung tissue from uninvolved areas of the lung from patients with pulmonary fibrosis suggests an analogous “field of injury” may be present in these patients [15, 16]. Accordingly, we undertook a study to compare the transcriptome of the nasal epithelium from patients with IPF compared with a set of age-matched controls. We observed consistent changes in gene expression suggesting upregulation of inflammatory pathways in the nasal epithelium of patients with IPF compared with controls. These findings support further studies of the nasal transcriptome to identify biomarkers that can identify patients with or at risk for IPF earlier in their disease.

## Patients and methods

### Study population

Approval for this study was obtained by the institutional review boards at Northwestern University (Chicago, IL, USA) and Instituto Nacional de Enfermedades Respiratorias Ismael Cosio Villegas (INER; Mexico City, Mexico). Patients and controls were explained about the study and signed a consent letter. Nasal mucosal biopsy procedures took place at a single center (INER). A total of 10 subjects (1 female, 9 male) meeting criteria for definite IPF [1] underwent nasal curettage. All IPF patients were clinically stable and without apparent viral or bacterial infection when the nasal epithelial cells were obtained. A total of 24 age-matched control subjects without a history of respiratory disease underwent biopsy. All biopsies were performed in an outpatient setting. The demographic characteristics of the enrollees are listed in Table 1. Patients and controls were unrelated individuals with Mexican-Mestizo ancestry and long-time residency in Mexico City. No differences were found in age and cigarette smoke exposure.

**Table 1:** Demographic and functional characteristics of enrollees

Variable	IPF Patients (*n* = 10)	Controls (*n* = 23)	*p*
Age (years)	68 ± 8.6	64.4 ± 8.6	0.2
Gender (male:female)	9:1	8:15	0.009
Smoking (never:former)	2:8	9:14	0.2
FVC (% predicted)	60.6 ± 14.7	95.7 ± 8.3	< 0.0001
DL_CO_ (% predicted)	36.3 ± 8.3	112.4 ± 18.8	< 0.0001
Saturation at rest (%)	94.8 ± 2.3	95.3 ± 1.9	0.2
Saturation post exercise (%)	85.4 ± 6.2	94 ± 4	0.0006

Abbreviations: *FVC* forced vital capacity, *DL*_*CO*_ diffusing capacity of the lungs for carbon monoxide

### Nasal curettage

Nasal epithelial cells were obtained by mucosal scrape biopsy of the inferior turbinate using a sterile plastic curette (Rhino-Pro curette, Arlington Scientific) as previously described to obtain a predominantly epithelial cell population [17, 18]. A total of 5 single-pass biopsies were performed per subject. Curettes were discarded if gross blood was visible on the curette. The curette tips were cut and placed into RNase-free collection tubes containing 200 μL of MagMAX cell lysis buffer and 2-mercaptoethanol, vortexed vigorously, and stored at − 80 °C. RNA was isolated using the commercially available MagMAX 96 extraction kit (ThermoFisher Scientific) adapted for the Bravo automated liquid handling platform (Agilent Technologies).

### Library preparation

RNA sequencing was performed at the RNA-Seq Center at the Division of Pulmonary and Critical Care, Feinberg School of Medicine, NU. Following RNA extraction, the RNA integrity number (RIN) was measured using the Agilent TapeStation 4200 (Additional file 1: Figure S1A and S1B). RNA-seq libraries were prepared using a QuantSeq 3′ mRNA-seq kit (Lexogen). Fragment size distribution for the libraries was assessed using the TapeStation 4200. Libraries were multiplexed and sequenced on the NextSeq 500 platform (Illumina) to an average depth of 7 × 10^6^ single-end reads. FASTQ files were processed using the QuantSeq 3′ mRNA-seq pipeline implemented on the Bluebee genomic platform (Bluebee) with the following steps: files were trimmed with BBDuk and aligned with STAR to the human genome (GRCh38.77), and a table of gene counts was generated from aligned reads with HTSeq. A MultiQC report [19] was created to evaluate RNA sequence quality (non-normalized data are shown in Additional file 2**:** Table S1, and normalized counts are shown in Additional file 3: Table S2, S3 and S4, and Additional file 4: Table S5).

### Differential expression analysis

Different pipelines using R version 3.4 [20] with Bioconductor version 3.6 [21], were used to select differentially expressed genes. Non-expressed genes were removed, those that had more than five reads in at least two samples for each gene were selected, and different normalization approaches using RUV (remove unwanted variation) as in Risso et al. [22] were tested. EdgeR [23, 24] and DESeq, which uses negative binomial distribution and a shrinkage estimator for the distribution’s variance [25], were used for estimating differentially expressed transcripts, and those genes with common results between the different methods were chosen. Gene annotations were implemented through biomaRt [26, 27]. Venn diagramming was performed using UpSetR [28].

### Functional analysis

We used the ToppGene suite, a portal for gene list enrichment analysis and candidate gene prioritization based on functional annotations and protein interaction networks to examine molecular functions and biological processes/pathways from the differentially expressed genes [29]. Enrichr, a gene set enrichment analysis web server, was used to compare our data with upregulated genes from signatures of microbe perturbations processed from GEO and to visualize top enriched terms using Clustergrammer [30].

## Results

### Baseline characteristics

The study involved 10 IPF patients and 24 age-matched control subjects. One control sample was removed from downstream analyses due to the low number of total reads and lower mapping rate. The average time of symptoms before IPF diagnosis was 28 + 16 months, and all of them had pulmonary impairment with a mean forced vital capacity of 60% predicted and a mean diffusing capacity of the lungs for carbon monoxide of 36% predicted at the time of the study. Seven patients were receiving pirfenidone, two were receiving nintedanib, and one of them had no treatment when samples were collected. Table 1 shows the demographic and functional characteristics of the enrollees.

### Transcriptomic profiling of the nasal epithelial Cells from IPF patients identifies differentially expressed genes

**Fig. 1:**
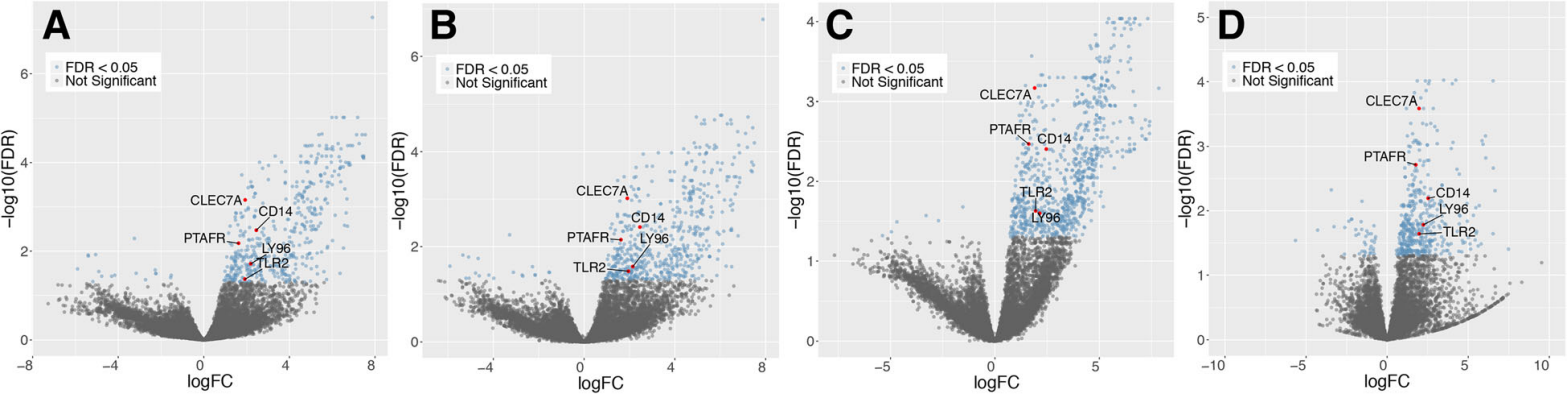
Volcano plots obtained with four pipelines. Pipelines: **a** EdgeR glmLRT normalization, **b** EdgeR glmLRT normalization with RUV, **c** EdgeR glmQLF normalization with RUV, **d** DESeq Wald normalization with RUV. Differentially expressed genes between control subjects and IPF patients are shown in blue (FDR *q* <  0.05). The upregulated genes associated with pattern-recognition receptor (PRR) molecular function are highlighted in red

**Fig. 2:**
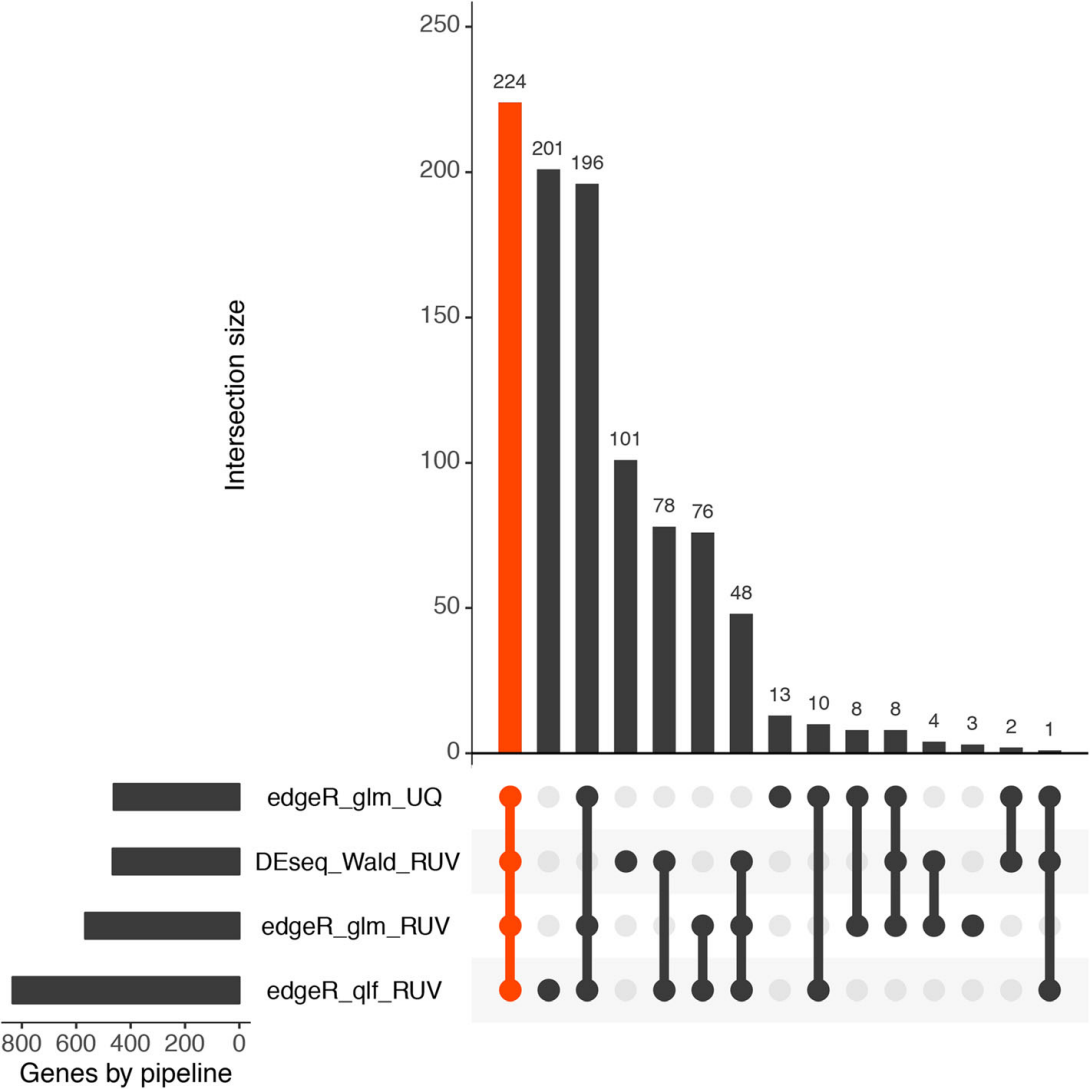
UpSet plot showing overlapping genes identified from the four pipelines (horizontal bar). Each column represents shared genes between the pipelines (linked dots)

We used two methods (EdgeR and DESeq) to compare gene expression changes in the nasal epithelium from patients with IPF and controls. The data were normalized using upper quartile or RUV, which gave us four different pipelines. We then selected the differentially expressed genes that were shared by all of them and passed the false discovery rate (FDR) cutoff of < 0.05 (Fig. 1). Most of the differentially expressed genes (222) were upregulated, and only two were downregulated in patients with IPF (Fig. 2; Additional file 5: Table S6). The ToppGene suite was used to generate a report of gene ontologies (GOs) related to molecular gene functions. As shown in Table 2, genes differentially expressed between controls and patients with IPF were associated with pattern-recognition receptor (PRR) functions, receptor activity, binding to the major histocompatibility complex (MHC), peptide antigen binding, and enzyme and cytokine binding.

**Table 2:** Gene ontologies: Molecular functions of differentially expressed genes

ID	Name	*p*-value	*q*-value, Bonferroni	*q*-value, FDR B&H	*q*-value, FDR B&Y	Hit Count in Query List	Hit Count in Genome	Hit in Query List
GO:0038187	pattern recognition receptor activity	5.52E− 07	3.76E− 04	1.88E− 04	1.34E− 03	5	17	CLEC7A, LY96, PTAFR, CD14, TLR2
GO:0008329	signaling pattern recognition receptor activity	5.52E−07	3.76E−04	1.88E− 04	1.34E−03	5	17	CLEC7A, LY96, PTAFR, CD14, TLR2
GO:0001875	lipopolysaccharide receptor activity	9.92E−06	6.75E−03	2.25E− 03	1.60E− 02	3	5	LY96, PTAFR, TLR2
GO:0042497	triacyl lipopeptide binding	1.02E−04	6.95E− 02	1.39E− 02	9.87E− 02	2	2	TLR1, TLR2
GO:0016230	sphingomyelin phosphodiesterase activator activity	1.02E−04	6.95E−02	1.39E− 02	9.87E− 02	2	2	STX4, NSMAF
GO:0042277	peptide binding	1.24E−04	8.44E−02	1.41E− 02	9.99E− 02	11	278	HLA-B, HLA-E, FFAR4, NFKBIA, TAP1, NUP98, PPIF, FURIN, TLR1, TLR2, SLC7A5
GO:0016004	phospholipase activator activity	2.07E−04	1.41E−01	1.92E−02	1.36E−01	3	12	STX4, CCL3, NSMAF
GO:0042287	MHC protein binding	2.54E−04	1.73E−01	1.92E−02	1.36E−01	4	31	LILRB2, HLA-E, CLEC7A, TAP1
GO:0042605	peptide antigen binding	2.88E−04	1.96E−01	1.92E−02	1.36E−01	4	32	HLA-B, HLA-E, TAP1, SLC7A5
GO:0033218	amide binding	3.09E−04	2.11E−01	1.92E−02	1.36E−01	11	309	HLA-B, HLA-E, FFAR4, NFKBIA, TAP1, NUP98, PPIF, FURIN, TLR1, TLR2, SLC7A5
GO:0005102	receptor binding	3.11E−04	2.12E−01	1.92E−02	1.36E−01	31	1601	RELN, HLA-B, LILRB2, HLA-E, ETS2, CLEC7A, LRG1, ADM, CMTM6, TYROBP, TMC8, CCL3, CCL4, FGR, TAP1, PLSCR1, NSMAF, SECTM1, PROK2, SELPLG, ICAM1, CCL3L3, SH2B2, NAMPT, GNA13, TNFSF13B, TLR1, TLR2, IRS2, LYN, IL1RN
GO:0060229	lipase activator activity	3.38E−04	2.30E−01	1.92E−02	1.36E−01	3	14	STX4, CCL3, NSMAF
GO:0019899	enzyme binding	4.15E−04	2.83E−01	2.17E−02	1.54E−01	35	1929	LILRB2, RNF19B, TNFRSF14, GBP1, PTAFR, CKB, SERPINB9, STX4, FGD4, CXCR4, XPO6, PLIN5, NFKBIA, EHD1, FGR, PLEK, NOS1, PLSCR1, ALOX5AP, SELL, RICTOR, LCP1, SH2B2, ZFP36, RHOH, LMNB1, FURIN, PPP1R18, IRS2, TNFAIP3, TNFRSF1B, LYN, TRIB1, MARCKS, SOD2
GO:0023029	MHC class Ib protein binding	5.98E−04	4.07E−01	2.91E−02	2.07E−01	2	4	LILRB2, TAP1
GO:0070891	lipoteichoic acid binding	9.90E−04	6.74E−01	4.49E−02	3.19E−01	2	5	CD14, TLR2
GO:0042288	MHC class I protein binding	1.17E−03	7.97E−01	4.74E−02	3.37E−01	3	21	LILRB2, HLA-E, TAP1
GO:0042802	identical protein binding	1.18E−03	8.07E−01	4.74E−02	3.37E−01	26	1359	CEBPD, DDX58, B2M, GBP1, BCL2A1, TYROBP, BNIP3L, GBP5, DGAT2, PLIN5, NFKBIA, GLUL, GCA, CCL3, CCL4, PLEK, NOS1, TAP1, ALOX5AP, RILPL2, LCP1, SH2B2, IFIT3, NAMPT, FTL, SOD2

### Biological processes and specific pathways

**Fig. 3:**
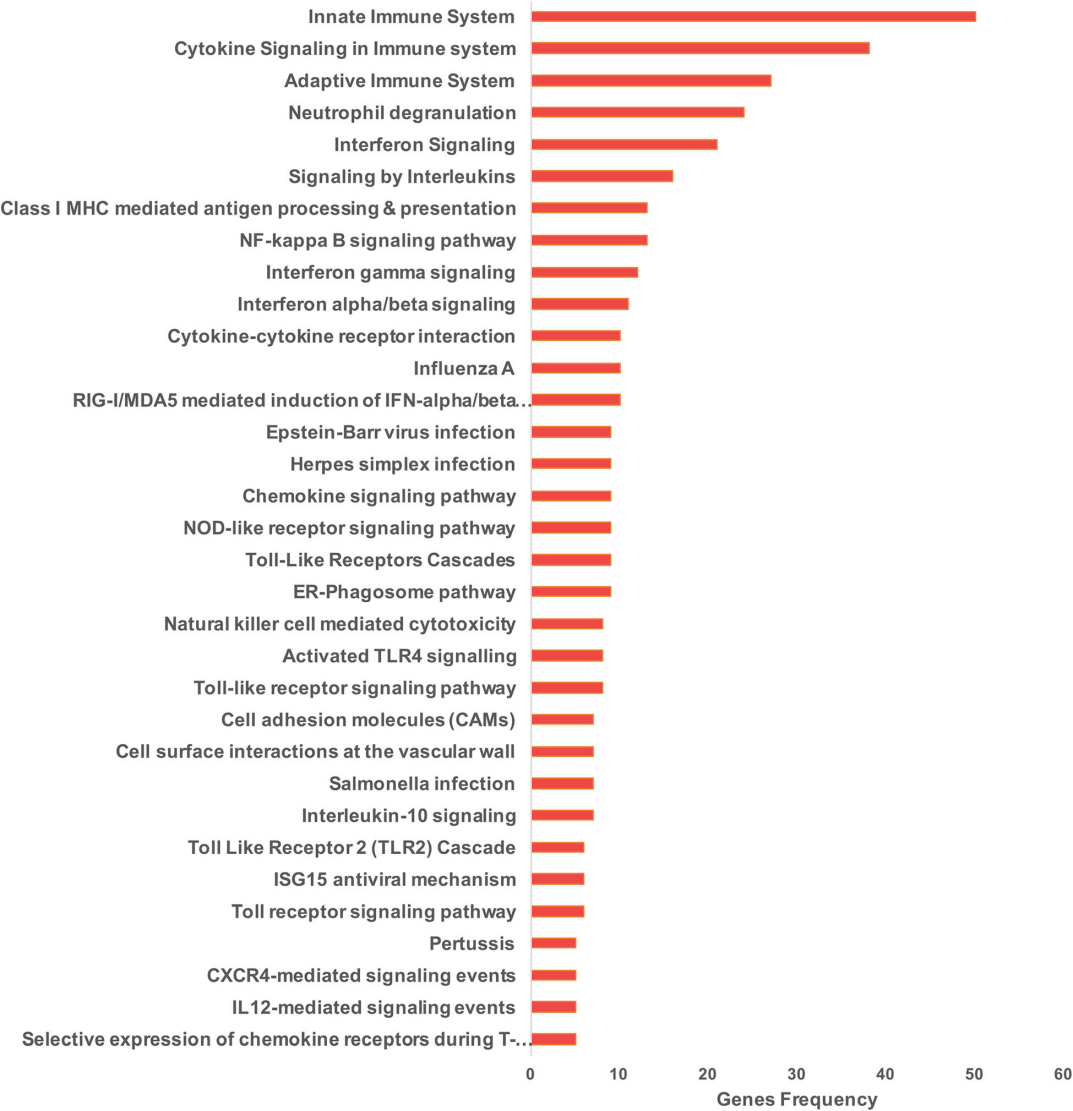
Selected signaling pathways obtained by the ToppGene suite. See Additional file 3: Table S3 for the complete list

The ToppGene suite was also used to generate biological processes and specific pathways from the differentially expressed genes. Overrepresented biological processes included immune response, defense response, response to external biotic stimulus, cytokine production, leukocyte and lymphocyte activation, and response to bacteria and virus (Additional file 6: Table S7). Regarding specific pathways, as shown in Fig. 3 and Additional file 6: Table S8, primarily signaling pathways related to innate and adaptive immune system, interferons (alpha, beta, and gamma), neutrophil degranulation, nuclear factor κB (NF-κB) signaling pathway, ER-phagosome pathway, Toll-like receptor cascades, antigen presentation, chemokine signaling pathway, and response to bacteria and influenza A virus were overrepresented. Taken together, these findings reveal a coordinated expression pattern consistent with the activation of immune and inflammatory defense mechanisms against bacteria and virus infection.

**Fig. 4:**
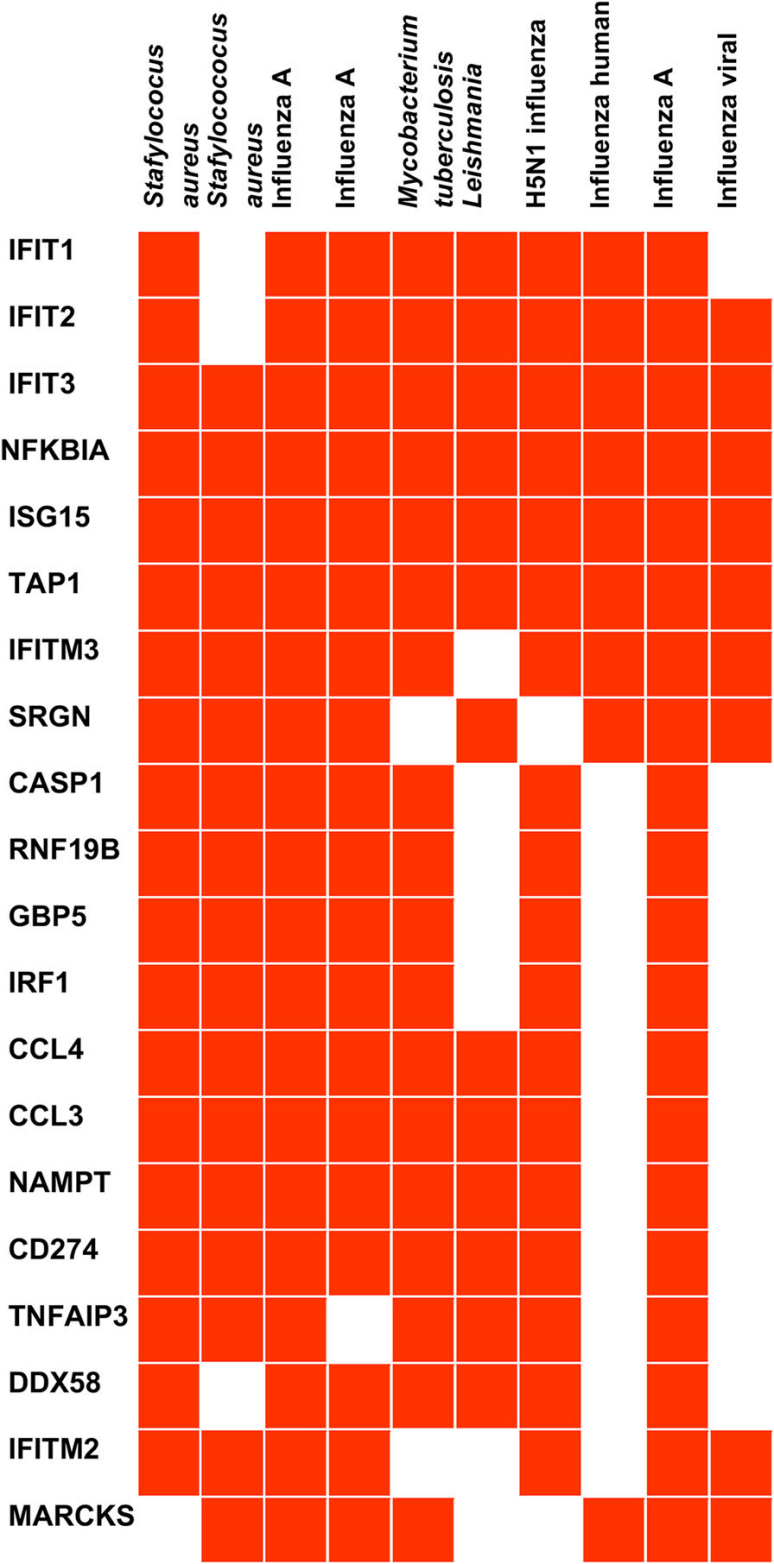
Clustergram of enriched terms showing upregulated gene enrichment using Microbe Perturbations from GEO. *Staphylococcus aureus* human monocyte-derived macrophages GDS4931 microbe:62 (adjusted *p*-value 4.004 × 10^− 54^); *Staphylococcus aureus* human macrophage GDS4931 microbe:60 (adjusted *p*-value 1.413 × 10^− 41^); Influenza A mouse lung 4 days post-infection GSE57452 microbe:311 and microbe: 310 (adjusted *p*-value 8.992 × 10^− 37^ and adjusted *p*-value 6.882 × 10^− 36^); *Mycobacterium tuberculosis* human THP-1 macrophages GDS4781 microbe:224 (adjusted *p*-value 7.781 × 10^− 33^); *Leishmania major* human dendritic cells GDS5086 microbe:150 (adjusted *p*-value 4.298 × 10^− 30^); H5N1 influenza virus human macrophage GDS3595 microbe:93 (adjusted *p*-value 1.5333 × 10^− 31^); Influenza human whole blood GDS57452 microbe:312 (adjusted *p*-value 1.361 × 10^− 29^); Influenza A mouse lung 5 post-infection GSE57452 microbe:312 (adjusted *p*-value 3.042 × 10^− 29^); Influenza virus human whole blood GDS3919 microbe:45 (adjusted *p*-value 6.551 × 10^− 28^)

Accordingly, we found using an enrichment analysis tool (Enrichr), that 20 of the upregulated genes were also found in the gene set library Microbe Perturbations from GEO [24]. Our shared genes include those observed in human macrophages infected with *Staphylococcus aureus,* in mouse lungs infected with influenza A virus*,* in dendritic cells following infection with *Leishmania major,* in macrophages infected with the virulent strain H37Rv of *Mycobacterium tuberculosis,* and in primary human macrophages after infection with influenza A (H5N1) virus ([31–35]; Fig. 4).

Given the disparity in gender (90% male in the IPF group, and 35% in the control group), we analyzed whether some of the found differences could be attributed to gender. For this purpose, we compared in the control group the global gene expression of males and females, and we did not find significant differences according to gender in any of the genes or pathways that were dysregulated in IPF (Additional file 7: Table S9). Moreover, using the DESeq with Wald RUV approach, we compared IPF males versus control males, and many of the genes and the biological processes that differentiate IPF versus controls were preserved.

Likewise, we wondered whether smoking exposure, an important environmental risk, influenced our findings. For this purpose, we compared the transcriptome of IPF former smokers versus controls former smokers using the DESeq with Wald RUV approach, and also found that most of the genes and ontology terms that differentiate IPF versus controls remained (Additional file 8: Table S10).

Finally, to identify a simplified nasal epithelial gene signature, we examined the most highly upregulated genes across the cohort (FDR cutoff of < 0.005 and fold change > 3, in all methods used) and obtained a list of 12 genes that were upregulated in most of the patients keeping most of the revealed biological processes (Additional file 9: Table S11).

## Discussion

IPF is a devastating and destructive lung disease of unknown etiology and unclear pathogenesis. Unbiased genome wide association studies in patients with pulmonary fibrosis and targeted genomic studies in patients with a family history of IPF have identified rare and common variants in genes that encode proteins expressed in the airway and alveolar epithelium that incur an increased risk of developing disease [36–39]. Because the onset and progression of symptoms in patients with IPF is insidious, even patients with these known risk factors often present with late stage disease. High resolution computed tomography can be diagnostic for IPF and can detect early disease; however, its utility as a screening is limited by its cost and risk for radiation exposure. Accordingly, safe and inexpensive tests that can be serially performed to identify patients early in their disease are needed.

Transcriptomic profiling of the nasal epithelium for disease diagnosis is an attractive alternative to profiling of the whole lung tissue obtained via biopsy, or bronchial epithelium obtained via bronchoscopy. This approach assumes that a regional epithelial abnormality in the lung is a consequence of changes in gene expression in epithelial cells throughout the respiratory tract, often described as a “field of injury”. Nasal transcriptomic profiling has already demonstrated its utility in detecting patients with lung cancer, cystic fibrosis, and chronic obstructive pulmonary disease, and identifying disease endotypes in asthma [40–44].

In this study, we observed consistent differences in the nasal transcriptome of patients with IPF compared with age-matched healthy controls. Upregulated pathways included interferon signaling, cytokine signaling in the immune system, neutrophil degranulation, NF-κB signaling pathway, interferon gamma signaling, and interferon alpha/beta signaling. Our results are consistent with those described by Luzina and colleagues, who observed a similar increase in the expression of inflammatory genes in macroscopically “normal” regions (although with microscopic signs of lung damage) of lung explants from patients with IPF [16]. Further supporting the concept of a field effect, Pankratz et al. observed that a machine learning tool applied to transcriptomic data from transbronchial biopsies performed equally well as a classifier of UIP compared with other fibrotic pathologies irrespective of the amount of alveolar tissue in the biopsy [15].

Upregulated genes in the nasal epithelium of patients with IPF did not include genes previously implicated in the pathogenesis of the disease. As the nasal epithelium is not pathologically abnormal in patients with pulmonary fibrosis, this result is perhaps unsurprising. The upregulation of inflammatory genes may reflect a nonspecific response to abnormalities in the distal lung. In support of this hypothesis, a similar inflammatory signature was observed in the transcriptome of lung tissue distant from the primary tumor in patients with lung cancer [45]. It is possible, however, that factors more directly related to the pathobiology of pulmonary fibrosis induce the upregulation of inflammatory genes. Supporting this point of view, a recent study using single-cell RNAseq to distinguish the transcriptional profiles of epithelial subtypes in IPF from healthy lungs, corroborated the profound loss of normal epithelial cell identities and interestingly, it demonstrated that upregulated pathways in IPF included chemokine signaling pathway, leukocyte transendothelial migration, bacterial invasion of epithelial cells and natural killer cell-mediated cytotoxicity [46]. Moreover, in our own dataset of patients with pulmonary fibrosis in which we sequenced whole lung tissue, flow sorted alveolar type II cells and alveolar macrophages through single cell RNA-Seq, although we did not see significant overlap in specific genes identified in previous studies of whole lung tissue, we observed the upregulation of pathways involved in inflammatory processes (available in preprint form https://www.biorxiv.org/content/early/2018/04/06/296608).

The factors that might influence this immune/inflammatory response include changes in the respiratory microbiome [47–49] or respiratory viral infections [50, 51], both of which have been suggested to contribute to the progression of the disease. Thus for example, progression of IPF has been associated with the presence of specific members within the Staphylococcus and Streptococcus genera [47]. Likewise, in a comprehensive analysis of host-microbiome interaction in which peripheral blood gene signature, lung microbial community, and IPF outcomes were integrated, it was shown that changes in the lung microbiome was associated with the induction of immunologic signaling pathways which in turn was significantly associated with poorer progression-free survival [52].

Interestingly, neither gender nor smoking seem to influence our results.

Our study has several important limitations, most importantly the small sample size of our cohorts. Furthermore, our nasal sequencing studies used 3′ mRNA-seq, precludes analysis of differences in isoform expression and non-coding RNA molecules, and largely precludes analysis of bacterial or viral transcripts. Furthermore, our depth of sequencing in the nasal transcriptome was low. In addition, only one of the IPF patients in the nasal transcriptome study was therapy naïve, and it is possible that some of the changes we saw represent effects of treatment. The finding of robust gene expression changes despite these limitations strongly supports further investigation of this approach in larger, longitudinal studies with deeper sequencing. These studies could be paired with analysis of the nasal microbiome and examination of epithelial RNA for viral and bacterial transcripts.

## Conclusion

In summary, this feasibility study indicates consistent differences in the nasal transcriptome from patients with IPF and age-matched healthy controls. As nasal sampling is fast, nearly painless and inexpensive, these findings support further research to explore the utility of the gene expression in the nasal epithelium as a biomarker for the identification of patients with pulmonary fibrosis. Moreover, in our small dataset, we were able to identify a small number of genes that were consistently upregulated in patients with IPF compared with controls. If validated in larger cohorts of IPF patients, upregulation of a small number of genes might be used to identify patients for more comprehensive screening (e.g. low dose CT).

In addition, many patients with interstitial lung abnormalities are identified on screening CTs for lung cancer or other reasons, and there are no markers that distinguish which of these patients are at increased risk for progressive disease [53]. Even if the inflammatory gene signature we identified is not related directly to disease pathobiology, a non-invasive biomarker that could identify patients with interstitial lung abnormalities incidentally found on CT at increased risk for progression to IPF would be clinically valuable.

The finding that genes involved in inflammation are upregulated in the nasal epithelium of patients with fibrosis suggests pairing of nasal transcriptome measurements with simultaneous measures of the nasal bacterial and viral DNA microbiome may be informative. These future studies should take advantage of the non-invasive aspects of this test, which allows serial measurements in patients at increased risk for developing fibrosis, or monitoring of patients who are initiating antifibrotic therapy.

## Additional files

Additional file 1: **Figure S1.** (A) Box plot of the RNA integrity number equivalent (RINe) showing distribution of IPF versus control samples. (B) Box plot of the RNA yield showing distribution of IPF versus control samples. (TIF 1813 kb)

Additional file 2: Non-normalized data gene counts. (CSV 1995 kb)

Additional file 3: Normalized counts using methods edgeR UQ (**Table S2**), edgeR UQ with RUV and RNA control (**Table S3**), and edgeR glmLRT with RUV and empirical control (**Table S4**). (ZIP 2119 kb)

Additional file 4: Normalized counts using DESeq wald with RUV. (CSV 5188 kb)

Additional file 5: **Table S6.** Differentially expressed genes obtained using DESeq and EdgeR. (ZIP 36 kb)

Additional file 6: **Table S7.** Gene Ontology: Biological Processes obtained with Toppgene Suite. **Table S8.** Pathways obtained by Toppgene Suite. (ZIP 79 kb)

Additional file 7: **Table S9. a** Differentially expressed genes (Control Group Male vs Female) obtained using EdgeR glmLRT normalization. **b** Differentially expressed genes (Control Group Male vs Female) obtained using EdgeR glmLRT normalization with RUV. (XLS 105 kb)

Additional file 8: **Table S10.** Differentially expressed genes obtained using DESeq Wald with RUV (IPF smokers vs CTRL smokers). (XLS 106 kb)

Additional file 9: **Table S11.** Simplified gene signature. (XLS 59 kb)

## Abbreviations

FDA: Federal Drug Administration
FDR: False discovery rate
GEO: Gene expression omnibus
GOs: Gene ontologies
HRCT: High resolution computed tomography
IPF: Idiopathic pulmonary fibrosis
MHC: Major histocompatibility complex
mRNA-seq: 3′ messenger RNA sequencing
NF-κB: nuclear factor κB
PRR: Pattern-recognition receptor
RUV: Remove unwanted variation
STAR: Spliced transcript alignment to a reference
UIP: Usual interstitial pneumonia
