# Supplementary figures and images for: Inflammatory pathways are upregulated in the nasal epithelium in patients with idiopathic pulmonary fibrosis
Source: Respir Res. 2018 Nov 26;19:233. doi: 10.1186/s12931-018-0932-7 (PMC6257973; doi:10.1186/s12931-018-0932-7)

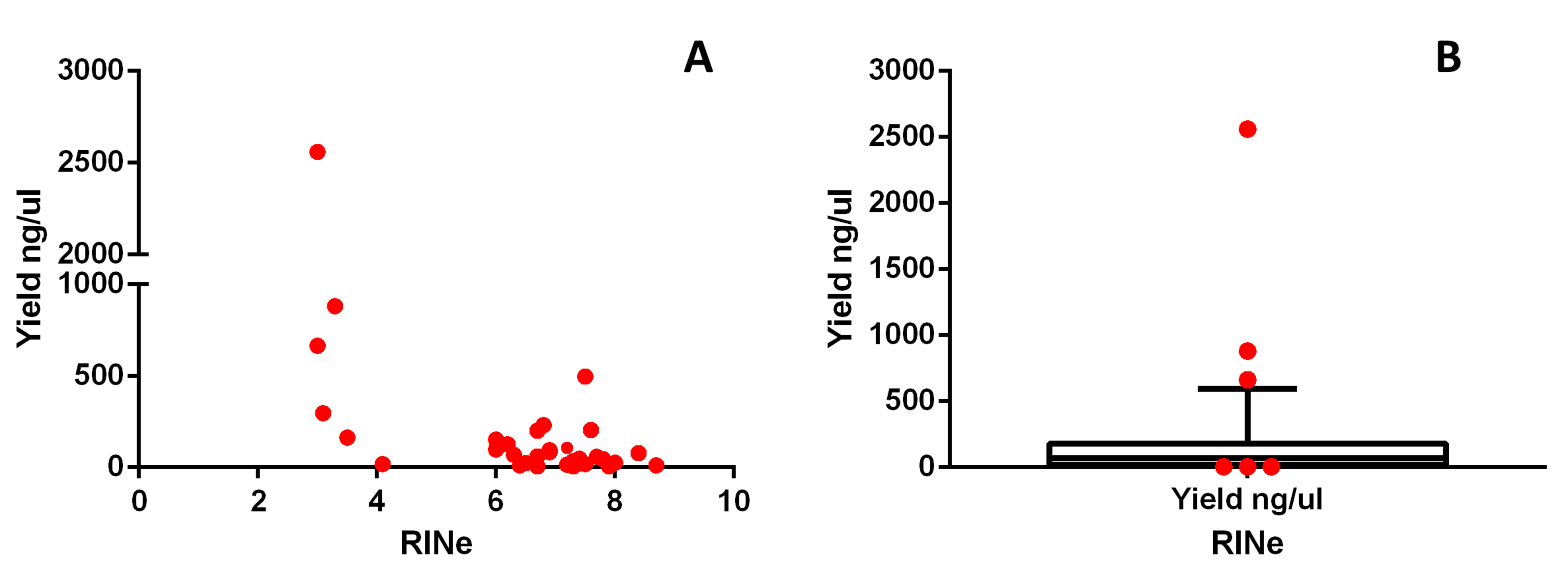

Supplement: Supplementary file 1 — Figure S1. (A) Box plot of the RNA integrity number equivalent (RINe) showing distribution of IPF versus control samples. (B) Box plot of the RNA yield showing distribution of IPF versus control samples. (TIF 1813 kb) [file 12931_2018_932_MOESM1_ESM.tif]
